# Aetiology and use of antibiotics in pregnancy-related infections: results of the WHO Global Maternal Sepsis Study (GLOSS), 1-week inception cohort

**DOI:** 10.1186/s12941-024-00681-8

**Published:** 2024-02-24

**Authors:** Carolina C. Ribeiro-do-Valle, Mercedes Bonet, Vanessa Brizuela, Edgardo Abalos, Adama Baguiya, Fernando Bellissimo-Rodrigues, Mihaela Budianu, Lucian Puscasiu, Marian Knight, David Lissauer, Catherine Dunlop, Shevin T. Jacob, Sadia Shakoor, Luis Gadama, Bouchra Assarag, João Paulo Souza, Jose G. Cecatti, the WHO GLOSS research group, Mohammad Iqbal Aman, Bashir Noormal, Virginia Díaz, Marisa Espinoza, Julia Pasquale, Charlotte Leroy, Kristien Roelens, Griet Vandenberghe, M. Christian Urlyss Agossou, Sourou Goufodji Keke, Christiane Tshabu Aguemon, Patricia Soledad Apaza Peralta, Víctor Conde Altamirano, Rosalinda Hernández Muñoz, José Guilherme Cecatti, Carolina C. Ribeiro-Do-Valle, Vincent Batiene, Kadari Cisse, Henri Gautier Ouedraogo, Cheang Kannitha, Lam Phirun, Tung Rathavy, Elie Simo, Pierre-Marie Tebeu, Emah Irene Yakana, Javier Carvajal, María Fernanda Escobar, Paula Fernández, Lotte Berdiin Colmorn, Jens Langhoff-Roos, Wilson Mereci, Paola Vélez, Yasser Salah Eldin, Alaa Sultan, Abdulfetah Abdulkadir Abdosh, Alula M. Teklu, Dawit Worku Kassa, Richard Adanu, Philip Govule, Charles Noora Lwanga, William Enrique Arriaga Romero, María Guadalupe Flores Aceituno, Carolina Bustillo, Rigoberto Castro, Bredy Lara, Vijay Kumar, Vanita Suri, Sonia Trikha, Irene Cetin, Serena Donati, Carlo Personeni, Guldana Baimussanova, Saule Kabylova, Balgyn Sagyndykova, George Gwako, Alfred Osoti, Zahida Qureshi, Raisa Asylbasheva, Aigul Boobekova, Damira Seksenbaeva, Faysal El Kak, Saad Eddine Itani, Sabina Abou Malham, Meilė Minkauskienė, Diana Ramašauskaitė, Owen Chikhwaza, Eddie Malunga, Haoua Dembele, Hamadoun Sangho, Fanta Eliane Zerbo, Filiberto Dávila Serapio, Nazarea Herrera Maldonado, Juan Ismael Islas Castañeda, Tatiana Caraus, Ala Curteanu, Victor Petrov, Yadamsuren Buyanjargal, Seded Khishgee, Bat-Erdene Lkhagvasuren, Amina Essolbi, Rachid Moulki, Nafissa Bique Osman, Zara Jaze, Arlete Mariano, Hla Mya Thway Einda, Thae Maung Maung, Khaing Nwe Tin, Tara Gurung, Amir Babu Shrestha, Sangeeta Shrestha, Kitty Bloemenkamp, Marcus J Rijken, Thomas Van Den Akker, María Esther Estrada, Néstor J. Gómez Pavón, Olubukola Adesina, Chris Aimakhu, Bukola Fawole, Rizwana Chaudhri, Saima Hamid, M. Adnan Khan, María del Huatuco PilarHernández, Nelly M. Pimentel Zavaleta, Maria Lu Andal, Carolina Paula Martin, Zenaida Dy Recidoro, Mihaela-Alexandra Budianu, Lucian Puşcaşiu, Léopold Diouf, Dembo Guirassy, Philippe Marc Moreira, Miroslav Borovsky, Ladislav Kovac, Alexandra Kristufkova, Sylvia Cebekhulu, Laura Cornelissen, Priya Soma-Pillay, Vicenç Cararach, Marta López, María José Vidal Benedé, Hemali Jayakody, Kapila Jayaratne, Dhammica Rowel, Mohamed Elsheikh, Wisal Nabag, Sara Omer, Victoria Tsoy, Urunbish Uzakova, Dilrabo Yunusova, Thitiporn Siriwachirachai, Thumwadee Tangsiriwatthana, Aquilino M. Pérez, Jhon Roman, Gerardo Vitureira, Dinh Anh Tuan, Luong Ngoc Truong, Nghiem Thi Xuan Hanh, Mugove Madziyire, Thulani Magwali, Stephen Munjanja, Mónica Chamillard, Bukola Fawole, Seni Kouanda, Pisake Lumbiganon, Ashraf Nabhan, Ruta Nadisauskiene, Linda Bartlett, Shevin T. Jacob, Khalid Yunis, Liana Campodónico, Cristina Cuesta, Hugo Gamerro, Daniel Giordano, Fernando Althabe, A. Metin Gülmezoglu

**Affiliations:** 1 Department of Gynaecology and Obstetrics, Faculty of Medical Sciences, University of Campinas, R. Alexander Fleming, 101, Campinas, São Paulo, CEP 13083-888 Brazil; 2 Department of Sexual and Reproductive Health and Research, UNDP/UNFPA/UNICEF/WHO/World Bank Special Programme of Research, Development and Research Training in Human Reproduction (HRP), WHO, Geneva, Switzerland; 3 Centro Rosarino de Estudios Perinatales (CREP), Rosario, Argentina; 4 Kaya Health and Demographic Surveillance System (Kaya-HDSS), Research Institute of Health Sciences (IRSS), Ouagadougou, Burkina Faso; 5 Ribeirão Preto Medical School, Ribeirão Preto, Brazil; 6 George Emil Palade University of Medicine, Pharmacy, Science, and Technology of Târgu Mureş, Târgu Mureş, Romania; 7 National Perinatal Epidemiology Unit, Nuffield Department of Population Health, University of Oxford, Oxford, OX3 7LF UK; 8 University of Liverpool, Blantyre, Malawi; 9 Institute of Life Course and Medical Sciences, University of Liverpool, Liverpool, UK; 10 University of Birmingham, Birmingham, UK; 11 Walimu, Mbarara, Uganda; 12 Department of Clinical Services, Liverpool School of Tropical Medicine, Liverpool, UK; 13 Department of Pathology and Laboratory Medicine, Aga Khan University, Karachi, Pakistan; 14 Department of Microbiology, Aga Khan University, Karachi, Pakistan; 15 Department of Obstetrics and Gynaecology, University of Malawi, Zomba, Malawi; 16 National School of Public Health, Rabat, Morocco; 17 Department of Social Medicine, Ribeirão Preto Medical School, Ribeirão Preto, Brazil

**Keywords:** Maternal sepsis, Maternal morbidity, Infections, Antibiotic

## Abstract

**Background:**

Pregnancy-related infections are important contributors to maternal sepsis and mortality. We aimed to describe clinical, microbiological characteristics and use of antibiotics by source of infection and country income, among hospitalized women with suspected or confirmed pregnancy-related infections.

**Methods:**

We used data from WHO Global Maternal Sepsis Study (GLOSS) on maternal infections in hospitalized women, in 52 low-middle- and high-income countries conducted between November 28th and December 4th, 2017, to describe the frequencies and medians of maternal demographic, obstetric, and clinical characteristics and outcomes, methods of infection diagnosis and causative pathogens, of single source pregnancy-related infection, other than breast, and initial use of therapeutic antibiotics. We included 1456 women.

**Results:**

We found infections of the genital (n = 745/1456, 51.2%) and the urinary tracts (UTI) (n = 531/1456, 36.5%) to be the most frequent. UTI (n = 339/531, 63.8%) and post-caesarean skin and soft tissue infections (SSTI) (n = 99/180, 55.0%) were the sources with more culture samples taken and microbiological confirmations. *Escherichia coli* was the major uropathogen (n = 103/118, 87.3%) and *Staphylococcus aureus* (n = 21/44, 47.7%) was the commonest pathogen in SSTI. For 13.1% (n = 191) of women, antibiotics were not prescribed on the same day of infection suspicion. Cephalosporins (n = 283/531, 53.3%) were the commonest antibiotic class prescribed for UTI, while metronidazole (n = 303/925, 32.8%) was the most prescribed for all other sources. Ceftriaxone with metronidazole was the commonest combination for the genital tract (n = 98/745, 13.2%) and SSTI (n = 22/180, 12.2%). Metronidazole (n = 137/235, 58.3%) was the most prescribed antibiotic in low-income countries while cephalosporins and co-amoxiclav (n = 129/186, 69.4%) were more commonly prescribed in high-income countries.

**Conclusions:**

Differences in antibiotics used across countries could be due to availability, local guidelines, prescribing culture, cost, and access to microbiology laboratory, despite having found similar sources and pathogens as previous studies. Better dissemination of recommendations in line with antimicrobial stewardship programmes might improve antibiotic prescription.

**Supplementary Information:**

The online version contains supplementary material available at 10.1186/s12941-024-00681-8.

## Background

Infection can cause or contribute to maternal deaths and severe maternal morbidity [1, 2]. Pregnancy-related infections (direct obstetric complications, such as chorioamnionitis, endometritis, urinary tract, obstetric surgical wound, and breast, are responsible for 10.7% of maternal deaths according to the World Health Organization (WHO)’s latest estimates [1, 3, 4]. The Global Burden of Disease study estimated that more than 12 million cases of maternal sepsis and other pregnancy-related infections occurred in 2017 [3]. A systematic review and meta-analysis on pregnancy-related infections and maternal sepsis estimated 39 chorioamnionitis, 16 endometritis, 12 wound infections, and 0.5 sepsis cases per 1000 women giving birth [4]. However, the contribution of infections to maternal mortality and morbidity could be even higher, as these estimates do not include deaths and morbidity due to abortion-related infections or non-pregnancy-related infections that could be exacerbated by pregnancy (indirect obstetric complications), such as pneumonia, flu, COVID-19, malaria, vector-borne and other neglected diseases.

Results from the multi-country Global Maternal Sepsis Study (GLOSS) [5] suggested that 70.4 pregnant or recently pregnant women per 1000 livebirths admitted or already hospitalized present with maternal infection (direct or indirect complication), with large variations between regions: from 38.6 in high-income countries (HIC) to 106.4 in upper-middle-income countries (U-MIC). GLOSS also showed that the most common sources of maternal infections were those of the urinary tract (27.9%), genital tract (endometritis (15.1%), chorioamnionitis (14.9%)) and abortion-related uterine infection (8·5%), skin or soft tissues (14.8%) and respiratory tract (9%) [5]. Other studies reporting on the source of infection causing maternal sepsis conducted in HIC also show that genitourinary and respiratory tract are the most common infection sources among pregnant or recently pregnant women [6–10].

Published data on causative pathogens of maternal infections have been scarce, particularly from low- and middle-income countries (LMIC). Studies in the UK and the USA have shown that more than half of women with maternal infection do not get a microbiological confirmation or identification of a causative pathogen [7, 9, 11, 12]. When identified, causative agents were mostly Gram-negative including mainly *Escherichia coli* [9, 11, 12].

Adequate use of antimicrobials for prevention and treatment of infections are key towards achieving Sustainable Development Goals (SDG) targets, for reduction of infection-related maternal and newborn deaths and tackling antimicrobial resistance (AMR) [13]. For this, early initiation of appropriate antimicrobial drugs, based on accurate diagnosis of the source of infection and probable causative agent is needed, and can improve survival [14, 15]. WHO has published recommendations on prevention and treatment of postpartum infection [15] and the AWaRe (Access, Watch, Reserve) tool to guide antibiotic prescription and prevent the promotion of antimicrobial resistance [16]. This is particularly important in LMIC where antimicrobial resistance (AMR) rates are high, data on maternal infections sources and pathogens are scarce and antibiotic availability may vary [17, 18]. Insights on antimicrobial prescription patterns to treat maternal infections in health facilities would help understand the compliance to these recommendations [19].

How pregnancy-related infections are diagnosed and treated is still not completely known in different settings, especially in LMIC. Therefore, we used data from GLOSS to describe maternal demographic, obstetric, and clinical characteristics and outcomes, methods of infection diagnosis and causative pathogens, by source of pregnancy-related infection, as well as initial use of therapeutic antibiotics by source and country income level.

## Methods

### Study design and participants

**Fig. 1:**
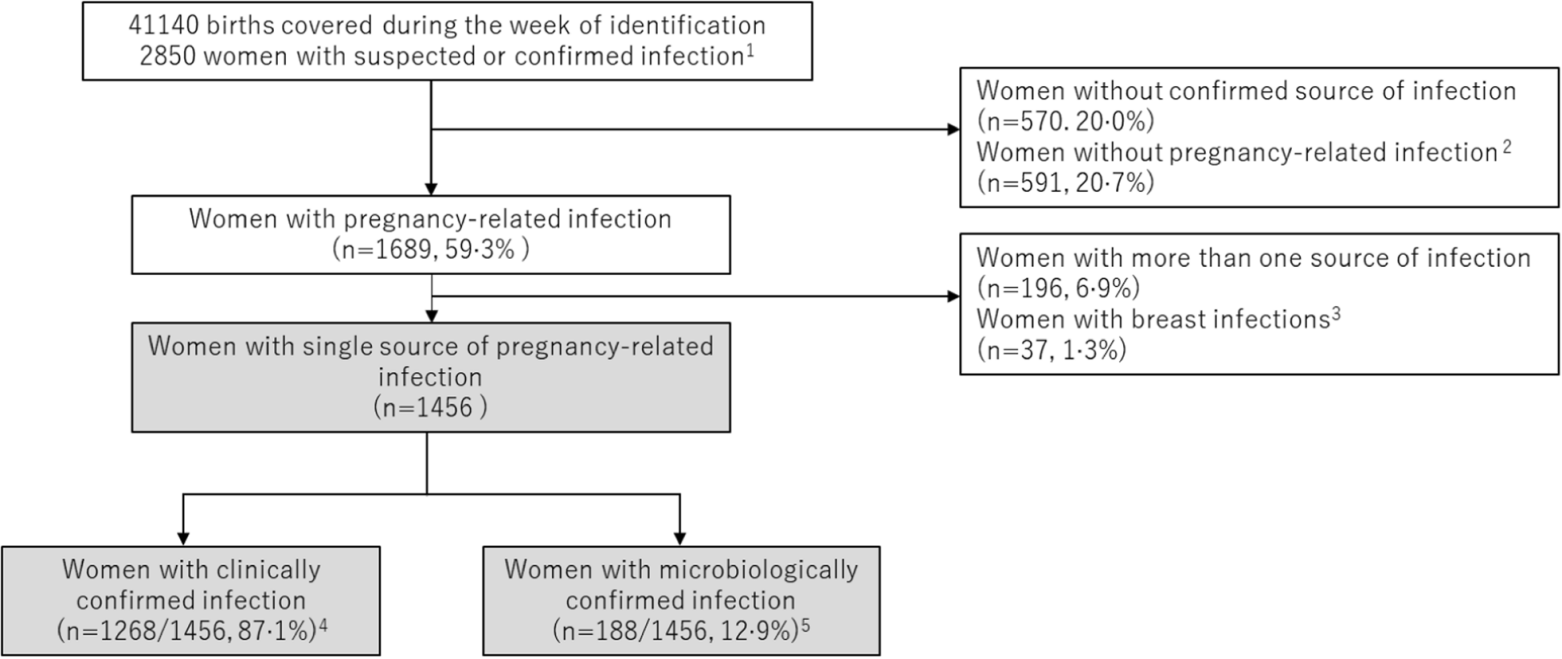
Study population flowchart. Percentages are shown as n of total sample. 1. GLOSS study population; 2. Women with other infections (respiratory, bloodstream, peritonitis or abdominal cavity, central nervous systems, unspecified); 3. Breast infections were excluded due to small numbers; 4. Clinically confirmed refers to infections confirmed only by clinical examination, imaging or laboratory; 5. Microbiologically confirmed refers to infections with identified pathogen with either by Gram staining or other type of microscopy finding, any matching positive cultures of any body fluid. A match to the source of infection was a case where the specimen for the source of the positive culture sample (taken at any time) was considered adequate for the diagnosis of that infection

GLOSS was a facility-based, prospective, 1-week inception cohort study implemented in 713 health care facilities in selected geographical areas in 52 low- (LIC), lower-middle- (L-MIC), upper-middle (U-MIC) and high-income countries (HIC), (Additional file 1: Fig. S1). The full protocol [20] and main results of the study [5] are fully described elsewhere. Briefly, during a seven-day period, from November 28th, until December 4th, 2017, 2850 pregnant or recently pregnant women admitted to or already hospitalized in participating facilities with suspected or confirmed infection were included in the study (Fig. 1). The fact that the study was conducted in fall in the Northern hemisphere and in spring in the Southern hemisphere was supposed to help to counterbalance the effect of seasonality that is always a concern for infectious diseases. Women were followed during their stay in health facilities until discharge, death or transfer to another health facility outside the study area. All data were collected through standardized paper forms from medical charts by local teams, without interaction with the women, and then fed into an online electronic platform especially built for the purpose of the study. The forms allowed reporting of multiple sources of infection, pathogens identified, and use of therapeutic antibiotics.

All identification data were confidentially stored by study coordinators. Written informed consent or a waiver of written consent was obtained as required by local or national committees. Opting-out was possible whenever decided by the participant. The protocol was approved by the WHO Ethics Review Committee (protocol ID: A65787, approved on 08/06/2017) and respective national and/or institutional ethics committees.

Out of the 2850 women included in the study, we excluded from this analysis 570 women for whom the primary source of the current infection was not confirmed, 591 with non-pregnancy-related infections, and 196 with more than one source of infection for whom it was difficult to investigate microbiological results and antibiotic use (Fig. 1). In addition, 37 women who had breast infections were excluded, due to the small numbers. Therefore, we analysed 1456 pregnant or recently pregnant women who had a confirmed single source of pregnancy-related infection, including urinary tract infection (UTI), chorioamnionitis, endometritis, skin and soft tissue infection (SSTI) after caesarean section and abortion-related uterine infection (ARUI).

For this analysis, we used data on maternal demographic (e.g.: age, schooling, country income level, number of previous births), clinical, and obstetric characteristics; source of infection, methods used to determine source of infection, use of antibiotic therapy (class and date of initiation), whether samples for culture were taken and their results, surgical management of the source of infection, length of stay in the facility, and admission and length of stay in intensive care unit (ICU).

We defined SSTI as post-caesarean surgical site infection occurring at least one calendar day after the procedure until 30 days post-surgery. We used the same definitions of infection severity as in a previous GLOSS [5] publication: less severe infections, infection with complications (invasive procedure to treat the source of infection (vacuum aspiration, dilatation and curettage, wound debridement, incision and drainage, percutaneous, culdotomy, laparotomy and lavage, other surgery, admission to intensive care or high dependency unit or transfer to another facility)), and infection-related severe maternal outcome (SMO: maternal near miss or death).

We defined the method of confirmation of infection as only clinical if based on clinical examination, general laboratory, radiologic findings or urinalysis alone without microbiological findings, or if the sample from which the pathogen was recovered did not match the source of infection. Microbiologically confirmed was defined as an infection confirmed with either Gram staining or other type of microscopy finding, any specific antigenic, serologic or molecular, and/or matching positive cultures of any body fluid, which comprised blood- and organ-related cultures (Additional file 1: Table S1). A match to the source of infection was identified when the specimen for the source of the positive culture sample (taken at any time) was considered adequate for the diagnosis of that infection (e.g., urine for UTI). A non-match was a case where the specimen was inadequate for the source of infection (e.g., urine for SSTI). The bacterial and fungal pathogens were reported on the genus or species level.

To report on the use of antibiotics, we considered the treatment prescribed on the same day of infection suspicion or diagnosis (at eligibility for entry into the study). We included in our analysis whether it was a single drug or a drug combination and if the treatment choice was aligned with WHO recommendations [15] (ampicillin with gentamicin for chorioamnionitis and clindamycin with gentamicin for endometritis and ARUI). We defined AMR according to local protocols and reported pathogens as non-susceptible to specific antimicrobial agents, as recommended by the Global Antimicrobial Resistance and Use Surveillance System (GLASS) [21].

### Statistical analysis

First, we described the distribution of maternal demographics, obstetric, and clinical characteristics, methods of infection diagnosis and causative pathogens, with respect to source of pregnancy-related infection. Then, we described the use of therapeutic antibiotics with respect to source of infection and country income level, using the 2018 World Bank classification [22]. Finally, we reported AMR for monomicrobial infections to avoid misclassification.

We performed sensitivity analyses by comparing the demographic, obstetric, and clinical characteristics of women included in this analysis with those who were excluded because of presenting multiple sources of infection. We found no significant differences between the two groups according to demographic or obstetric characteristics. However, women with one source of pregnancy-related infection who presented with less severe infection-related outcomes were less likely to require admission to ICU (p-value < 0.001) and had shorter ICU length of stay (p-value < 0.001) than those reported to have multiple sources of infection, with significant p-values. They had also fewer culture samples taken at any time (p-value = 0.02) but of those who did have cultures drawn, they were more likely to be before the administration of antibiotics (p-value = 0.01). A single source of pregnancy-related infection was more common than multiple sources of infection in HIC (p-value < 0.001) (Additional file 1: Table S2).

Data are presented as percentages when describing frequencies and median and interquartile ranges when describing continuous variables. Data explorations were performed to check distributions before choosing the appropriate tests to be used. The statistical significance was set at p < 0.05. The analyses were performed using STATA (StataCorp. 2019. Stata Statistical Software: Release 16. College Station, TX: StataCorp LLC).

### Role of the funding source

The funders of the study had no role in data collection, data analysis, data interpretation or writing of the report. The corresponding author had full access to all the data in the study and had final responsibility for the decision to submit for publication. The views of the funding bodies have not influenced the content of this manuscript.

## Results

### Sources of pregnancy-related infections

Among the 1456 women with one source of pregnancy-related infection included in this analysis, the most common pregnancy-related infection was UTI (531/1456; 36.5%), followed by chorioamnionitis (314/1456; 21.6%), endometritis (256/1456; 17.6%), SSTI (180/1456; 12.3%) and ARUI (175/1456; 12.0%).

A greater proportion of women, across the different sources of infections, lived with a partner, was between 19 and 35 years of age, was either nulliparous or had only one previous birth and, except for women with ARUI, had a high education level (Table 1). The majority of women were from L-MIC or LIC, except for women with UTI who mainly were from U-MIC. Amongst women with SSTI, we had data on both pre-operative skin preparation and antibiotic prophylaxis from 165 patients. Of those, 14 (8.5%) women received the first and 13 (7.9%) the latter. Most of the women with UTI (n = 447/531, 84.2%), chorioamnionitis (n = 225/314, 71.6%) and endometritis (n = 148/256, 57.8%) had less severe infections, whereas women with SSTI (n = 123/180, 68.3%) and ARUI (n = 142/175, 81.1%) presented mainly with complicated infections or infection-related SMO. SMO was found to be mainly associated with endometritis (n = 46/256, 18%) and ARUI (n = 37/175, 21%).

**Table 1:** Demographic, obstetric and clinical characteristics of women with single source of pregnancy-related infection, by source of infection (n = 1,456)

Characteristics	Urinary tract infection (n = 531)	Chorioamnionitis (n = 314)	Endometritis (n = 256)	Skin and soft tissue infection after caesarean section (n = 180)	Abortion-related uterine infection (n = 175)	p-value
	n (%)	n (%)	n (%)	n (%)	n (%)	
Age (years) (n = 1455)						0.004
< 19	64 (12.0%)	27 (8.6%)	26 (10.2%)	8 (4.4%)	15 (8.6%)	
19–35	414 (78.0%)	249 (79.3%)	190 (74.2%)	145 (80.6%)	125 (71.4%)	
> 35	53 (10.0%)	38 (12.1%)	40 (15.6%)	27 (15.0%)	35 (20.0%)	
Living with partner (yes -n = 1456)	430 (81.0%)	268 (85.4%)	222 (86.7%)	154 (85.6%)	117 (66.9%)	< 0.001
Schooling (years) (n = 1094)						0.002
≤ 11	195 (45.7%)	109 (44.5%)	67 (36.0%)	52 (40.6%)	65 (60.2%)	
> 11	232 (54.3%)	136 (55.5%)	119 (64.0%)	76 (59.4%)	43 (39.8%)	
Country income level (n = 1456)						< 0.001
Low-income	32 (6.0%)	44 (14.0%)	62 (24.2%)	57 (31.7%)	37 (21.1%)	
Lower-middle-income	197 (37.1%)	159 (50.6%)	110 (43.0%)	80 (44.4%)	99 (56.6%)	
Upper-middle-income	242 (45.6%)	60 (19.1%)	37 (14.4%)	25 (13.9%)	29 (16.6%)	
High-income	60 (11.3%)	51 (16.2%)	47 (18.4%)	18 (10.0%)	10 (5.7%)	
Number of previous births (n = 1444)						< 0.001
0–1	376 (71.4%)	231 (74.0%)	176 (69.8%)	131 (73.2%)	94 (54.0%)	
> 1	151 (28.6%)	81 (26.0%)	76 (30.2%)	48 (26.8%)	80 (46.0%)	
Location at the time of infection suspected or confirmed (n = 1456)						< 0.001
Arriving from home	314 (59.1%)	132 (42.2%)	103 (40.4%)	108 (60.3%)	104 (59.4%)	
Transferred from another facility	55 (10.4%)	54 (17.2%)	36 (14.1%)	11 (6.2%)	32 (18.3%)	
Already hospitalised	162 (30.5%)	127 (40.6%)	116 (45.5%)	60 (33.5%)	39 (22.3%)	
Severity of infection (n = 1456)						< 0.001
Less severe	447 (84.2%)	225 (71.6%)	148 (57.8%)	57 (31.7%)	33 (18.9%)	
Infection with complication^a^	51 (9.6)	62 (19.8)	62 (24.2)	109 (60.6)	105 (60.0)	
Infection-related severe maternal outcome^b^	33 (6.2)	27 (8.6)	46 (18)	14 (7.8)	37 (21.1)	
Admission to intensive or high dependency care (yes, n = 1336)	56 (11.3%)	34 (12.4%)	31 (13.8%)	16 (9.6%)	14 (8.1%)	0.404
Median length of stay in intensive care unit, days (IQR)	3 (2–6)	2 (1–5)	2 (1–10)	3 (1–5)	2 (2–5)	
Median length of stay in health facility, days (IQR)	5 (3–7)	4 (3–7)	7 (5–11)	11 (6–17)	4 (3–7)	

Data are n (%) or median (IQR: inter-quartile range); Country income according to 2018 World Bank classification

^a^Includes women who had an invasive procedure to treat the source of infection (vacuum aspiration, dilatation and curettage, wound debridement, drainage [incision, percutaneous, culdotomy], laparotomy and lavage, other surgery), admission to intensive care or high dependency unit, or transfer to another facility

^b^Infection-related maternal death or near-miss. Geographical areas in six western European countries (Belgium, Denmark, Italy, Spain, the Netherlands, the UK) did not collect data for WHO near-miss criteria

### Microbiological diagnosis

The majority of women in our sample had infections confirmed using only clinical methods, with low percentages of culture samples taken at any time, especially for the genital tract, mainly for ARUI (n = 56/175, 32.0%). Women had more culture samples taken at any time when UTI was the infection source (n = 339/531, 63.8%) and prior to antibiotic use (n = 242/531, 45.6%). Culture samples were taken prior to antibiotic initiation in 31·9% (n = 464/1456) of the cases, less so for ARUI (n = 27/175, 15.4%) (Table 2). Causative pathogens were more frequently identified in women with UTI (n = 126/531, 23.2%) and SSTI (n = 45/180, 25.0%), while all cases of endometritis were only clinically confirmed. Although 41·4% (n = 106/256) of women with endometritis had any sample collected for culture, most of them (n = 76/106, 71.7%) reported collection of samples not related to the specific source of infection, e.g. urine, and were classified as non-matching samples. Amongst microbiologically confirmed infections, bacterium was the predominant pathogen. *E. coli* was the most common uropathogen (n = 103/128, 80.4%), and *S. aureus* (n = 21/55, 11.7%) and *E. coli* (n = 12/55, 6.7%) were the most common pathogens in SSTI.

**Table 2:** Methods of diagnosis, pathogens and therapeutic antibiotics use among women with single source pregnancy-related infection, by source of infection (N = 1456)

Diagnostics and management	Urinary tract infection (N = 531)	Chorioamnionitis (N = 314)	Endometritis (N = 256)	Skin and soft tissue infection after caesarean section (N = 180)	Abortion-related uterine infection (N = 175)	p-value
	n (%)	n (%)	n (%)	n (%)	n (%)	
Method of confirmation of infection (n = 1456)						< 0.001
Clinically confirmed only^a^	408 (76.8%)	298 (94.9%)	256 (100.0%)	136 (75.6%)	170 (97.1%)	
Microbiologically confirmed	123 (23.2%)	16 (5.1%)	0 (0.0%)	44 (24.4%)	5 (2.9%)	
Any sample for culture drawn at any time (yes, n = 1448)	339 (63.8%)	109 (34.7%)	106 (41.4%)	99 (55.0%)	56 (32.0%)	< 0.001
Sample for blood culture drawn before administration of antibiotics (yes, n = 714)	242 (45.6%)	62 (19.7%)	74 (28.9%)	59 (32.8%)	27 (15.4%)	0.004
Non-matching culture samples to the source of infection^b^ (yes, n = 1456)	27/339 (7.9%)	22/109 (20.2%)	76/106 (71.7%)	8/99 (8.1%)	6/56 (10.7%)	< 0.001
Pathogen identified in matching samples by any methods^c^ (n = 258)						
Bacteria	118 (22.2%)	16 (5.0%)		44 (24.4%)	5 (2.9%)	
Monomicrobial	117 (22.0%)	13 (4.1%)		35 (19.4%)	4 (2.3%)	
Polymicrobial	1 (0.2%)	3 (0.9%)		9 (5.0%)	1 (0.6%)	
Fungi	8 (1.5%)	0 (0.0%)		1 (0.5%)	1 (0.6%)	
Bacteria and fungi identified^d^ (n = 258)						< 0.001
* Escherichia coli*	103 (80.4%)	8 (44.5%)		12 (21.8%)	2 (16.7%)	
* Klebsiella pneumoniae*	8 (6.3%)	4 (22.2%)		9 (16.4%)	0 (0.0%)	
* Staphylococcus aureus*	3 (2.3%)	5 (37.8%)		21 (38.2%)	1 (8.3%)	
Other gram-negative^e^	2 (1.6%)	1 (5.5%)		7 (12.7%)	7 (58.4%)	
Other gram-positive^f^	4 (3.1%)	0 (0.0%)		5 (9.1%)	1 (8.3%)	
* Candida spp*	8 (6.3%)	0 (0.0%)		1 (1.8%)	1 (8.3%)	
Therapeutic antibiotics started the day of suspicion or diagnosis of infection^g^ (n = 1456)						< 0.001
None	66 (12.4%)	21 (6.7%)	43 (16.8%)	41 (22.8)	20 (11.4%)	
Single antibiotic	329 (62.0%)	74 (23.6%)	45 (17.6%)	44 (24.4)	36 (20.6%)	
Antibiotic combination	136 (25.6%)	219 (69.7%)	168 (65.6%)	95 (52.8)	119 (68%)	
Most commonly prescribed therapeutic antibiotics at day of suspicion/diagnosis of infection						
Metronidazole	45 (8.5%)	126 (40.1%)	131 (51.2%)	69 (38.3%)	97 (55.4%)	
Cephalosporin 1st/2nd generation	126 (23.7%)	38 (12.1%)	36 (14.1%)	16 (8.9%)	17 (9.7%)	
Cephalosporin 3rd/4th generation	157 (29.6%)	94 (29.9%)	87 (34.0%)	53 (29.4%)	54 (30.9%)	
Aminoglycosides	58 (11.0%)	97 (30.9%)	81 (31.7%)	31 (17.3%)	47 (26.8%)	
Penicillin	6 (1.1%)	11 (3.5%)	5 (2.0%)	1 (0.6%)	1 (0.6%)	
Clindamycin	43 (8.1%)	41 (13.1%)	38 (14.8%)	21 (11.7%)	23 (13.1%)	
Co-amoxiclav	55 (10.0%)	67 (21.3%)	25 (9.8%)	20 (11.1%)	23 (13.1%)	

^a^Clinically confirmed refers to clinical examination, imaging, laboratory (without microbiological confirmation)

^b^Pathogen was identified in any body fluid not related to the source of infection

^c^Pathogen was identified with either by Gram staining or other type of microscopy finding or positive cultures of any body fluid matching the identified source of infection

^d^Each woman could have more than one type of microorganism identified

^e^Other gram-negative includes: *P. aeruginosa, A. baumannii, Citrobacter spp, Enterobacter spp, Proteus spp, Ureaplasma spp, Bacteroides spp*, and Enterobacterales

^f^Other gram-positive includes: Coagulase-negative Staphylococci*, Enterococcus spp* and *S. agalactiae*

^g^Same calendar day

### Antibiotic treatment

**Fig. 2:**
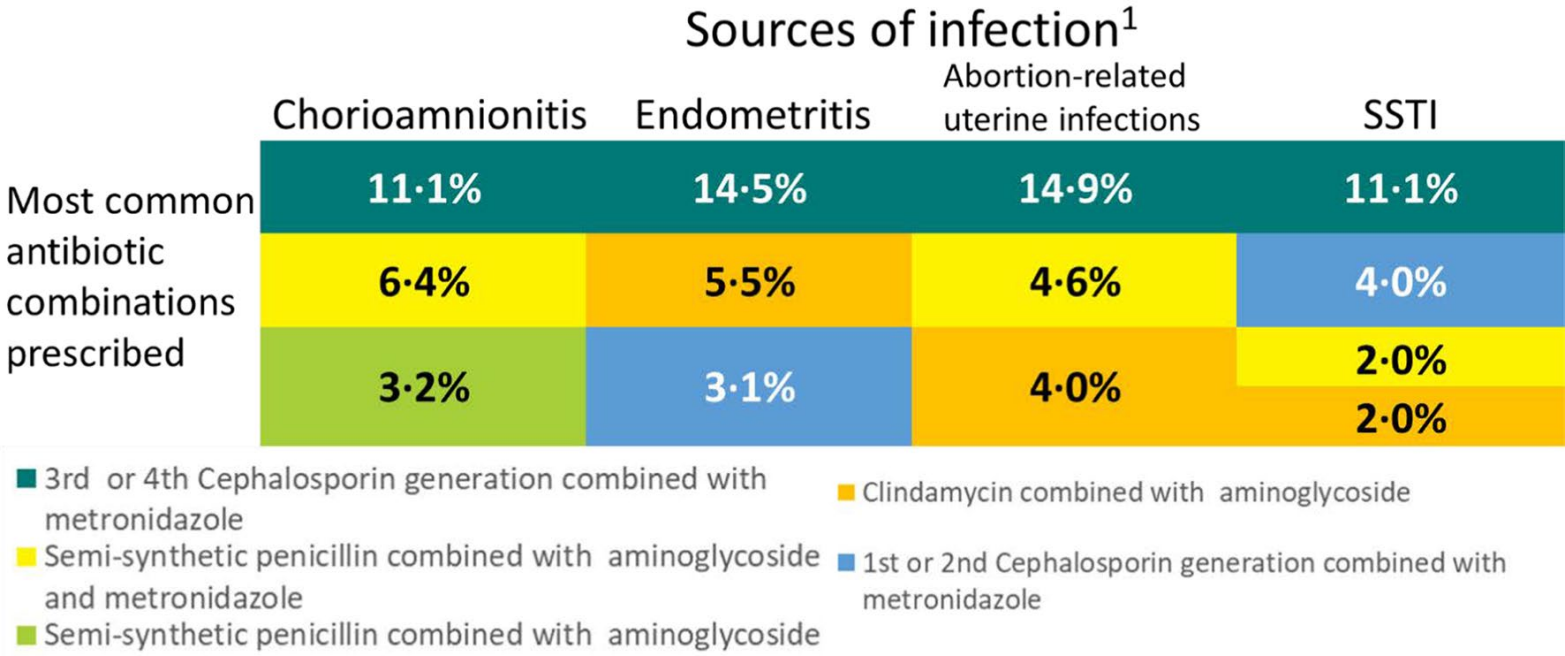
Most commonly antibiotic combinations prescribed among women with a single source of pregnancy-related infection, by source of infection. The first three most common combinations are shown. 1. UTI was excluded, for which prescription of combination of antibiotics is not recommended

**Fig. 3:**
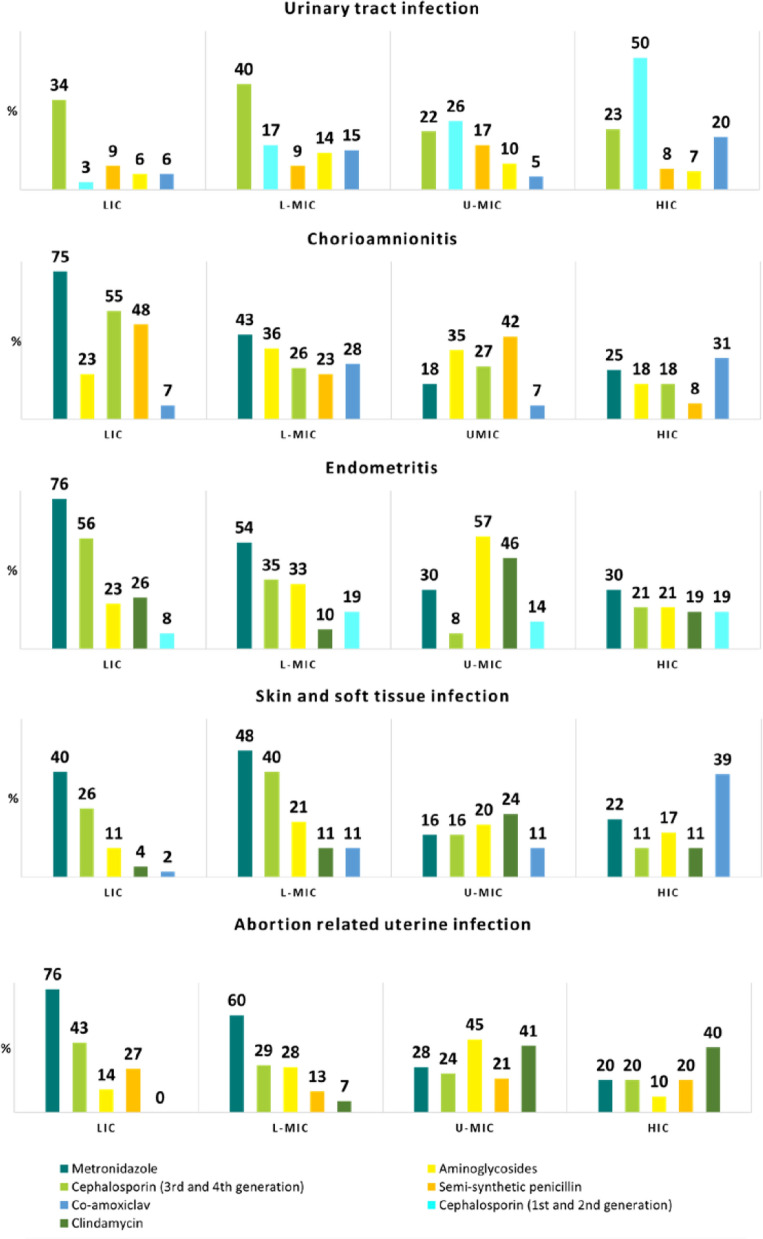
Most commonly prescribed antibiotics among women with one source of pregnancy-related infection, by source of infection and country income level. *LIC* low-income countries, *L-MIC* lower-middle-income countries, *UMIC* upper-middle income countries, *HIC* high-income countries (2018 World Bank classification) (N = 1456)

For 13.2% (n = 191/1456) of the women, no empiric antibiotic treatment was prescribed on the day of infection suspicion, ranging from 6·7% (n = 21/314) among those with chorioamnionitis to 22.8% of the women with SSTI (n = 41/180) (Table 2). Women with UTI received mainly single drug therapy (n = 329/531, 62.0%), whilst 64·9% (n = 601/925) of women with other sources of infections received mainly antibiotic combinations. The most prescribed antibiotic was metronidazole (chorioamnionitis: n = 126/341, 40.1%; endometritis: n = 131/256, 51.2%; SSTI: n = 69/180 (38.3%; ARUI: n = 97/175, 55.4%), except for UTI, where cephalosporins of third or fourth generations were more commonly used (n = 157/531, 29.6%). The most frequent combination of antibiotics prescribed was ceftriaxone and metronidazole for all genital tract infections (n = 98/745, 13.2%) and SSTI (n = 22/180, 12.2%) (Fig. 2). Only 3.2% (n = 10/314) of the women with chorioamnionitis and 5.5% (n = 14/256) and 4.0% (n = 7/175) of the women with endometritis and ARUI, respectively, were treated in accordance with the WHO recommendations on antibiotic treatment. Figure 3 presents the most commonly prescribed antibiotics on the day of infection suspicion/diagnosis, by source of pregnancy-related infection and country income. While metronidazole was the most prescribed drug in LIC, for both the genital tract (endometritis (n = 47/62, 76%), ARUI (n = 28/37, 76%) and chorioamnionitis (n = 33/44, 75%)) and SSTI (n = 23/57, 40%), in HIC practitioners preferred co-amoxiclav for chorioamnionitis (n = 16/51, 31%) and SSTI (n = 7/18, 39%) and first and second generation cephalosporins (n = 30/60, 50%) for UTI.

Antibiotic susceptibility tests (AST) were seldom reported. *Staphylococcus aureus* reported non-susceptibility to methicillin in 8 out of the 13 with reported AST results. Out of the 63 reported AST for *E. coli* isolates, ten (16%) were non-susceptible to aminoglycosides, 41 (65%) to ampicillin/amoxicillin, 17 (27%) to 3rd generation cephalosporins, three (5%) to carbapenems, ten (16%) to cotrimoxazole and 16 (25%) to fluoroquinolones (results not shown in tables due to small numbers).

## Discussion

We found infections of the urinary and genital tract to be the most common pregnancy-related infections among pregnant or recently pregnant women admitted for or already hospitalized with a single-source infection. UTI and SSTI were the sources with more culture samples taken and with more microbiological confirmations. *E. coli* was the most common uropathogen and *S. aureus* was the most frequent pathogen isolated in SSTI. On the day of infection suspicion, antibiotics were not prescribed for almost 15% of women. Cephalosporins were the most common antibiotic class prescribed for UTI, and metronidazole was for all other sources. Ceftriaxone with metronidazole was the most frequently used combination for genital tract and SSTI. Although AST data were infrequently available, we found that more than one-quarter of *E. coli* isolates from any source showed resistance to 3rd generation cephalosporins.

Our findings are similar to previous reports where genital [6–8, 10–12, 23, 24] and urinary tract [5] were the commonest sources of maternal infection during and after pregnancy. Most women with genital tract infections were from L-MIC or LIC, and women diagnosed with UTI mainly were from L-MIC and U-MIC. The fact that we found UTI to be the commonest source of pregnancy-related infection could be due to potential differences in hospital admission criteria. The smallest proportion of the sample was from HIC, nonetheless, almost a third of the HIC women had a UTI (similar to L-MIC). Only in U-MIC, more than 60% had a UTI. Women from middle-income countries (where 71.3% of our samples were collected) might have been admitted for treatment of a UTI, whereas women from LIC would have been treated as out-patients, either due to lack of resources or less diagnoses.

Though UTI was quite common, it was seldom complicated, whereas ARUI and SSTI had higher percentages of complications. This finding could be explained by the definition used for infections with complications that included having any intervention for treating the infection (e.g., curettage, drainage, and debridement). Endometritis and ARUI greater proportion of infected related SMO is a warning that uterus infections are of concern in this population. However, it could be also explained by poor adherence to infection prevention recommendations [15], such as antibiotic prophylaxis and limited access to safe abortion practices [25].

We found that blood culture samples were taken prior to antibiotic initiation in around only 30% of the cases, even less in ARUI (15.4%) despite solid recommendation [26]. Although there is scarce data on pathogens, our findings are similar to others from LIC [18] and HIC [9, 11, 12] in which *E. coli* was the major pathogen found. While Gram-negative bacilli, streptococci and *S. aureus* appear in varying numbers across obstetric populations, along with anaerobes, they are major causes of concern because of their contribution to potentially severe outcomes. In addition, it is more difficult to culture anaerobic bacteria, which requires complex laboratory support. Even in HIC, in studies that included only septic patients, less than two-thirds of women had a causative pathogen identified [9, 12]. Results from an HIC setting showed that in pregnancy-associated severe sepsis, as defined by authors in that study, microbiological results were reported in 35.3% and Gram-negative bacteria were the most frequently isolated pathogens [23]. Both the severity of the infection and access to a microbiology laboratory could explain these differences between settings. Blood cultures prior to initiating therapeutic antibiotics are recommended whenever assessing a patient with possible sepsis [26]. Lack of resources (that limits access to antibiotic treatments and laboratory services) and limited knowledge on what are the best and most effective ways to diagnose and treat infections may contribute to the high proportion of women who did not have culture samples taken. It was not possible to consider differences amongst countries due to the small amount (proportion) of identified pathogens.

We also found that culture samples were rarely obtained from the appropriate source given the suspected source of infection (e.g., obtaining a urine sample to investigate a genital tract infection). Considering that taking and processing a urine sample is much easier and cheaper, as compared to the uterus, amniotic fluid, and surgical wound, it is understandable that UTI has higher number of collected samples with microbiological confirmation. Nevertheless, timely collected blood culture samples, especially if anaerobic media is used, can contribute to microbiological diagnosis of pregnancy-related infections. Although, as we found in this analysis, it is seldom performed in clinical practise.

Almost 15% of women did not receive antibiotic treatment on the day of infection suspicion. Although availability of similar information is limited from other studies, we must stress the importance of the recommendation for immediate antibiotic treatment, whenever faced with the possibility of sepsis [26].

As was also found in a study in Tanzania [18] where ceftriaxone plus metronidazole was the commonest combination used to treat puerperal sepsis, we found the most prescribed antibiotic to be metronidazole with or without ceftriaxone for infections of the genital tract and SSTI. We also found very low adherence to WHO recommendations for antimicrobial treatment of peripartum infections, ampicillin plus gentamycin for chorioamnionitis and clindamycin plus gentamycin for endometritis [15]. WHO recommends the minimum indispensable and cheapest antibiotic, and therefore if there are more expensive antibiotics available which have less toxicity or easier administration (e.g., compared to gentamicin) and still appropriate microbiological specificity they might also be understood as correctly used.

We also found differences in prescription practices across country income (data not shown in tables). Perhaps the most important result of this study was that we found that the most prescribed drugs in HIC are in the Access group, according to AWaRe antibiotics categories, defined as drugs that should be used as the first or second choice of empiric therapy due to spectrum of activity and lower resistance induction potential [16]. In contrast, 3rd generation cephalosporins, one of the most prescribed antibiotics in LMIC, are in the Watch group which includes antibiotics with higher resistance potential that should be highly prioritized as key targets for antimicrobial stewardship. These recommendations are intended to guide and warrant best practices, and we urge them to be disseminated. Moreover, without routine use of culture and AST data to guide prescriptions, while the risk of overuse of these antibiotics is present, it is difficult to determine the appropriateness of empiric decisions. AMR data can be used to guide regional empiric choices, and diagnostic stewardship measures at facility level can support appropriate antibiotic use.

These above-mentioned differences can also be due to limited access to different classes of antibiotics [27], prompting the physician to prescribe wider spectrum of antibiotics to be on the safe side, or to lack of knowledge of spectrum and tissue penetration, or a combination of these factors. Nevertheless, this antibiotic use is a threat to limited resource settings in which microbiology access is restricted and AMR rates may be higher [17, 18].

To the best of our knowledge, this study is the first to assess sources of pregnancy-related infections, their causative agents and antibiotic treatment provided to hospitalised women in a wide range of low-, middle-, and high-income countries. Prior to this study, most of the data regarding the sources of infections and their causative pathogens came from HIC and among sepsis cases. In the present analysis, pregnancy-related infections were considered, and the severity was described. However, our study has limitations. As it was an observational study, the different infection source proportion throughout country income might be due to variability in local hospital admission criteria. In addition, limitations in the completeness of laboratory result reports, as well as lack of access to laboratories at all, might have led to fewer microbiological data. We also did not collect information on the duration of each antibiotic therapy or changes in therapeutic regimens during hospital stay; therefore, we were not able to describe treatment length or antibiotic adjustments. The fact that we had to exclude women with more than one source of infection lead us to analyse a group with lower severity, as shown in the sensitivity analysis (Additional file 1: Table S2).

Even though there are widely available WHO guidelines on prevention and treatment of postpartum infections, these were seldom followed, leading to either inadequate antibiotic treatment and potential increases in AMR. Changes in practice are needed, from raising awareness on the need of better tailored antibiotic treatment, AMR emergence and the safety of following WHO’s recommendations. Other studies focusing on aetiological aspects of pregnancy-related infections and AMR could generate evidence on therapeutic decision-making and on tailoring setting-specific recommendations and policies for the proper management of infections.

### Supplementary Information

**Additional file 1: Table S1.** Specimens considered to be adequate for diagnosis according to each source of infection. **Figure S1**. Countries included in the WHO Global Maternal Sepsis Study. **Table S2**. Baseline characteristics of the women with confirmed infection included in the study, according to the number of sources of infection reported

## Data Availability

The data used for this analysis can be made available upon reasonable request, in accordance with the GLOSS research group data sharing policy and WHO policy of data use and data sharing. For further information, contact the corresponding author.

## Abbreviations

AMR: Antimicrobial resistance
ARUI: Abortion related uterine infection
AST: Antimicrobial susceptibility test
AWaRe: Access, watch, reserve
GLASS: Global Antimicrobial Resistance and Use Surveillance System
GLOSS: Global Maternal Sepsis Study
HIC: High-income country
ICU: Intensive care unit
LIC: Low-income country
LMIC: Low- and middle-income countries
SDG: Sustainable development goals
SMO: Severe maternal outcomes
SSTI: Skin and soft tissue infection
UMIC: Upper middle-income country
UTI: Urinary tract infection
WHO: World Health Organization
