# Supplementary material for: Aetiology and use of antibiotics in pregnancy-related infections: results of the WHO Global Maternal Sepsis Study (GLOSS), 1-week inception cohort
Source: Ann Clin Microbiol Antimicrob. 2024 Feb 24;23:21. doi: 10.1186/s12941-024-00681-8 (PMC10894467; doi:10.1186/s12941-024-00681-8)
Supplement: Supplementary file 1 — Additional file 1: Table S1. Specimens considered to be adequate for diagnosis according to each source of infection. Figure S1. Countries included in the WHO Global Maternal Sepsis Study. Table S2. Baseline characteristics of the women with confirmed infection included in the study, according to the number of sources of infection reported [file 12941_2024_681_MOESM1_ESM.docx]

**Additional file 1**

**Table S1.** Specimens considered to be adequate for diagnosis according to each source of infection

| Source of infection | Adequate specimens |
| --- | --- |
| Chorioamnionitis | Blood, vaginal swab, endometrial swab, placenta and other than those specified in the form |
| Endometritis | Blood, vaginal swab, endometrial swab and other than those specified in the form |
| Abortion-related uterine infection | Blood, vaginal swab, endometrial swab, placenta and other than those specified in the form |
| Skin and soft tissue infection | Blood, wound swab, soft tissue and catheter |
| Urinary tract infection | Blood and urine |

**Table S2**. Baseline characteristics of the women with confirmed infection included in the study, according to the number of sources of infection reported

| **Baseline characteristics** | **Women with pregnancy–related infections** | | **p-value** |
| --- | --- | --- | --- |
|  | One source of pregnancy–related infections (n=1,493) | More than one source of infection (n=196) |  |
| **Age (years) (n =1,689)** |  |  |  |
| <19 | 146 (9·8%) | 20 (10·2%) | 0·95 |
| 19-35 | 1,150 (77·0%) | 149 (76·0%) |  |
| >35 | 197 (13·2%) | 27 (13·8%) |  |
| **Living with partner** | 1,223 (81·9%) | 166 (84·7%) | 0·52 |
| **Schooling (years) (n =1,275)** |  |  |  |
| **≤11** years | 498 (44·3%) | 80 (52·6%) | 0·05 |
| > 11 years | 625 (55·7%) | 72 (47·4%) |  |
| **Country income level** |  |  |  |
| Low-Income | 234 (15·7%) | 46 (23·5%) | <0·001 |
| Lower-Middle-Income | 655 (43·9%) | 97 (49·5%) |  |
| Upper-Middle-Income | 402 (26·9%) | 52 (26·5%) |  |
| High-Income | 202 (13·5%) | 1 (0·5%) |  |
| **Number of previous births (n=1,674)** |  |  |  |
| 0-1 | 1,040 (70·2%) | 129 (66·8%) | 0·33 |
| >1 | 441 (29·8%) | 64 (33·2%) |  |
| **Location at the time of infection suspected/diagnosed** |  |  |  |
| Arriving from home | 791 (53·1%) | 96 (49·0%) | 0·33 |
| Transferred from another facility | 191 (12·8%) | 32 (16·3%) |  |
| Already hospitalised | 507 (34·1%) | 68 (34·7%) |  |
| **Severity of infection** |  |  | <0·001 |
| Less severe | 938 (62·8%) | 81 (41·3%) |  |
| Infection with complication^1^ | 396 (26·5%) | 49 (25·0%) |  |
| Infection-related severe maternal outcome^2^ | 159 (10·7%) | 66 (33·7%) |  |
| **Sample for culture drawn at any time** | 724 (48·8%) | 113 (58·9%) | 0·02 |
| **Sample for culture drawn before administration of antibiotics** | 474 (65·0%) | 57 (50·4%) | 0·01 |
| **Admission to intensive or high dependency care (n=1,556)** | 151 (11·1%) | 57 (29.2%) | <0·001 |
| **Median length of stay in health facility, days (IQR)** | 7·5 (7·2-7·9) | 13.5 (10·9-16) | <0·001 |
| **Median length of stay in intensive or high dependency unit, days (IQR)** | 4·4 (3·4-5·2) | 12·9 (8·3-17·5) | <0·001 |

Data are n (%) or median (IQR); Country income according to 2018 World Bank classification; 1. Includes women who had an invasive procedure to treat the source of infection (vacuum aspiration, dilatation and curettage, wound debridement, drainage [incision, percutaneous, culdotomy], laparotomy and lavage, other surgery), admission to intensive care or high dependency unit, or transfer to another facility. 2. Infection-related maternal death or near-miss. Geographical areas in six western European countries (Belgium, Denmark, Italy, Spain, the Netherlands, the UK) did not collect data for WHO near-miss criteria

Figure S1: Countries included in the WHO Global Maternal Sepsis Study.


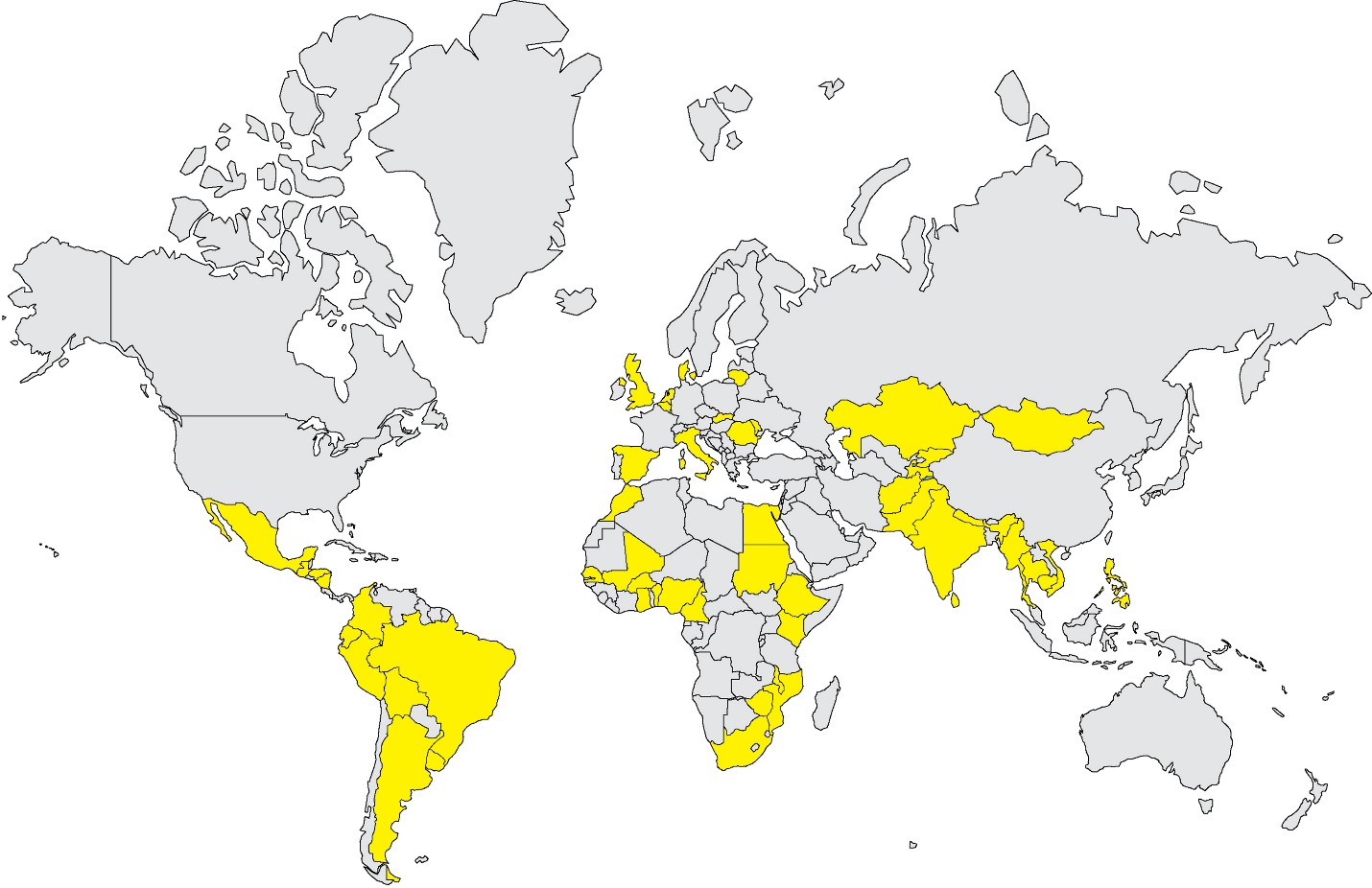


Countries coloured in yellow were included.
